# Aerobic oxidative synthesis of quinazolinones and benzothiazoles in the presence of laccase/DDQ as a bioinspired cooperative catalytic system under mild conditions†

**DOI:** 10.1039/c9ra10303a

**Published:** 2020-04-08

**Authors:** Nadia Ghorashi, Zahra Shokri, Reza Moradi, Amira Abdelrasoul, Amin Rostami

**Affiliations:** Department of Chemistry, Faculty of Science, University of Kurdistan 66177-15175 Sanandaj Iran a_rostami372@yahoo.com +988716624004 +989183730910; Department of Chemical and Biological Engineering, University of Saskatchewan 57 Campus Drive Saskatoon Saskatchewan S7N 5A9 Canada

## Abstract

The current study applied laccase/DDQ as a bioinspired cooperative catalytic system for the synthesis of quinazolinones (80–95% yield) and benzothiazoles (65–98% yield) using air or O_2_ as ideal oxidants in aqueous media at ambient temperature. The aerobic oxidative cyclization reactions occur in two steps: (i) chemical cyclization; (ii) chemoenzymatic oxidation. These methods are more environment-friendly, efficient, simple and practical than other reported methods due to the use of O_2_ as an oxidant, laccase as an eco-friendly biocatalyst, aqueous media as the solvent and free from any toxic transition metal and halide catalysts. Therefore, these methods can be applied in pharmaceutical and other sensitive synthetic procedures.

## Abstract 2

In this paper laccase/DDQ as a bioinspired cooperative catalytic system was applied for the synthesis of quinazolinones and benzothiazoles using air or O_2_ as ideal oxidant in aqueous media under mild conditions.

## Graphical abstract

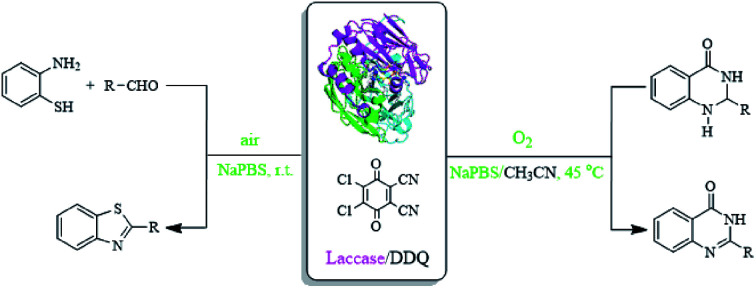

## 1. Introduction

Quinazolin-4(3*H*)-ones are important nitrogen-containing heterocycles which have various biological and medicinal activities, including antibacterial, anticancer, antimicrobial, antidiabetic, antifungal, anticonvulsant and antiallergy.^1,2^ The cyclization of *o*-anthranilamides with aldehydes followed by subsequent oxidation is the most convenient method for the synthesis of these valuable compounds.^3,4^

Benzothiazoles are also important members of the family of fused heterocycles that have attracted much attention because of their diverse biological activity and medical applications.^5^ The most popular approach to the synthesis of benzothiazoles is the condensation of 2-aminothiophenols with aldehydes under oxidative conditions.^6–8^

The reported procedures for the synthesis of quinazolinone and benzothiazole derivatives generally suffer from some drawbacks including the use of excess amounts of expensive oxidants, the formation of large amounts of toxic waste, harsh reaction conditions, and tedious work-up. Therefore, the development of a simple procedure, which is green and environmentally benign, for the synthesis of these valuable compounds is very important.

Quinones have been applied as oxidants in organic chemistry and hydride acceptors in biological processes.^9^ Among quinones, 2,3-dichloro-5,6-dicyano-1,4-benzoquinone (DDQ) is well-known as an effective and readily available oxidant for numerous organic transformations.^10–12^ In spite of the utility of DDQ as a stoichiometric oxidant, its high toxicity and cost, the isolation problem because of the concomitant by-product DDQH_2_ are the main issues associated with utilizing DDQ on a large scale. To overcome these disadvantages, a combination of the catalytic amount of DDQ and a less expensive co-oxidant that regenerate DDQ from its reduced hydroquinone form have been developed.^13–15^ Recently, the catalytic oxidation systems using catalytic amounts of DDQ and a co-catalyst in the presence of molecular oxygen as a terminal oxidant have attracted more attention.^16–18^ Although these procedures have been successfully applied in the field of aerobic oxidations, the development of an alternative method which is green and employing eco-friendly co-catalyst such as biocatalysts in combination with DDQ for the aerobic oxidation of organic compounds is in demand.

Laccases, highly attractive biocatalysts in modern organic synthesis, are easily available multicopper oxidases produced by numerous organisms, including fungi, plants, and prokaryotes. Laccase and laccase-mediated system catalyze the oxidation of various organic compounds in the presence of O_2_ as an electron acceptor and produce H_2_O exclusively as a by-product.^19^

In continuation of our study in the catalytic applications of laccase enzyme in the aerobic oxidation of organic compounds,^20^ herein, we report the aerobic oxidative synthesis of quinazolinones and benzothiazoles in the presence of laccase/DDQ catalyst system.

## 2. Results and discussions

**Scheme 1:**
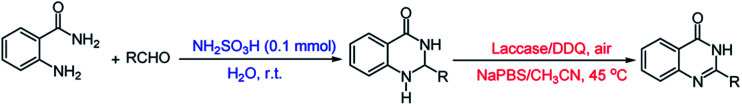
Aerobic oxidative cyclization synthesis of quinazolin-4(3*H*)-ones.

The oxidative cyclization synthesis of quinazolinones occurs in two-step sequence: (i) chemical cyclization of *o*-anthranilamide with aldehyde in the presence of sulfamic acid to afford 2,3-dihydroquinazolin-4(1*H*)-one (ii) chemoenzymatic aerobic oxidation of 2,3-dihydroquinazolin-4(1*H*)-one in the presence of laccase/DDQ catalyst system (Scheme 1).

Initially, the reaction of *o*-anthranilamide with benzaldehyde was chosen as a model system (Table 1). The formation of 2-phenyl 2,3-dihydroquinazolin-4(1*H*)-one was accomplished in the presence of sulfamic acid (0.1 mmol) at room temperature in H_2_O for 30 minutes. Subsequently, to optimize the reaction conditions for the aerobic oxidation of 2-phenyl-2,3-dihydroquinazolin-4(1*H*)-one to 2-phenyl quinazolin-4(3*H*)-one, the effects of the solvents, temperature and the amounts of laccase and DDQ were investigated (Table 1). Among the solvents, sodium phosphate buffer solution (NaPBS, 0.1 M, pH = 5)/CH_3_CN (4%) mixture had the highest isolated yield (Table 1, entry 4). The amounts of laccase and DDQ were also optimized. The results revealed that this transformation needs the double action of laccase and DDQ (Table 1, entries 1–6). However, the complete conversion of 2,3-dihydroquinazolin-4(1*H*)-one to desired product was observed in the presence of 200 U of laccase as co-catalyst, 0.2 mmol of DDQ as catalyst in NaPBS (0.1 M, pH = 5)/CH_3_CN (4%) mixture as solvent at 45 °C (Table 1, entry 4).

**Table 1:** Optimization of aerobic oxidation reaction conditions of 2-phenyl 2,3-dihydroquinazolin-4(1*H*)-one in the presence of laccase/DDQ catalyst system^a^

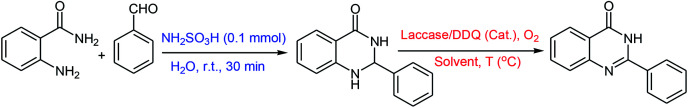					
Entry	DDQ (mol%)	Laccase (U)	Solvent	Temperature (°C)	Isolated yield%
1	-	200	MeCN/NaPBS	45	30^b^
2	5	200	MeCN/NaPBS	45	50^b^
3	10	200	MeCN/NaPBS	45	70^b^
4	20	200	MeCN/NaPBS	45	90^c^
5	20	100	MeCN/NaPBS	45	60^b^
6	20	—	MeCN/NaPBS	45	35^b^
7	20	200	MeOH/NaPBS	45	70^b^
8	20	200	NaPBS	45	45^b^
9	20	200	MeCN	45	—d
10	20	200	DMSO/NaPBS	45	—d
11	20	200	MeCN/NaPBS	60	40
12	20	200	MeCN/NaPBS	r.t.	60

a Reaction conditions unless stated otherwise: 2,3-dihydroquinazolin-4(1*H*)-one (1 mmol), O_2_ (balloon), phosphate buffer (0.1 M, pH 4.5, 12.5 mL), organic solvent (0.5 mL), 24 h.

b The reaction was not completed.

c The reaction was completed (conversion: 100%).

d No reaction.

The scope of this procedure was further examined by treating a number of substituted benzaldehydes with *o*-anthranilamide under optimized reaction conditions (Table 2). The results in Table 2 show that aromatic aldehydes containing electron-donating (methyl and methoxy) and electron-withdrawing (fluoro and bromo) groups were efficiently converted to the respective products in very good to excellent yields (Table 2, entries 1–9). It was also observed that the present method was equally applicable to the oxidative cyclization of terephthalaldehyde as a bifunctional aromatic aldehyde (Table 2, entry 10). It should be noted that in cases of 4-fluoro benzaldehyde and terephthalaldehyde very small amounts of starting material were observed together with the main product.

**Table 2:** Aerobic oxidative synthesis of quinazolin-4(3*H*)-one derivatives in the presence of laccase/DDQ catalytic system^a^

Entry	Substrate	Product	Time (h)	Isolated yield%	Mp. (°C) (lit.)
1	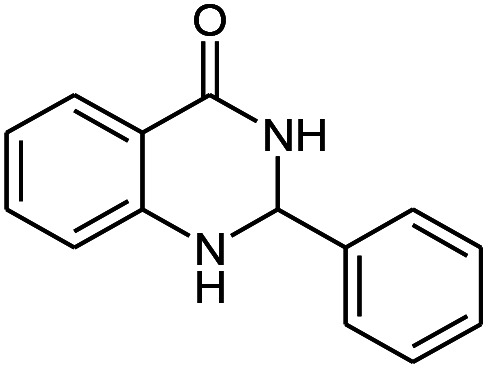	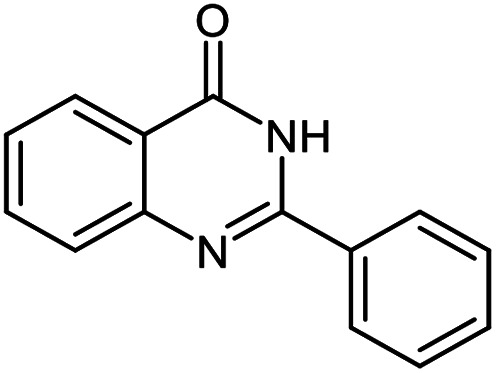	24	90	236–238 (ref. 21)
2	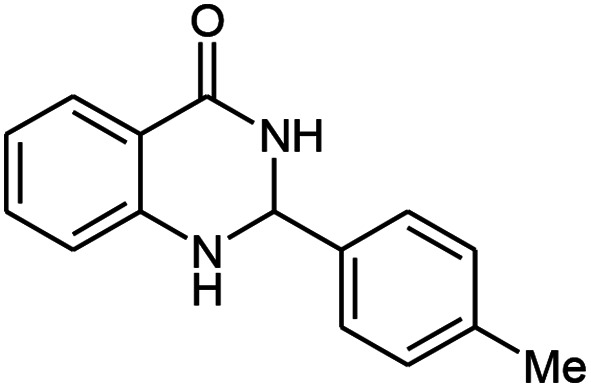	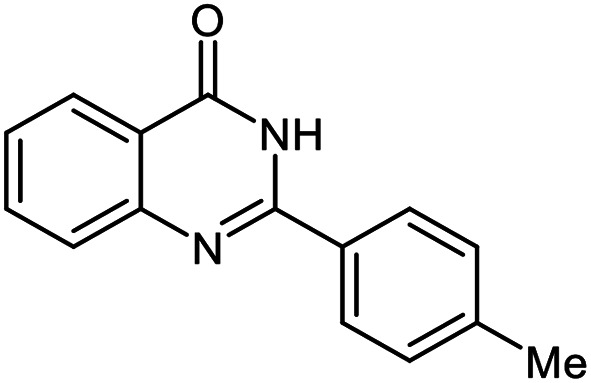	20	92	238–240 (ref. 21)
3	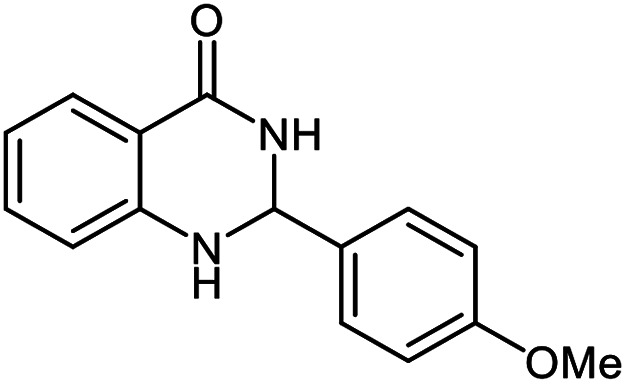	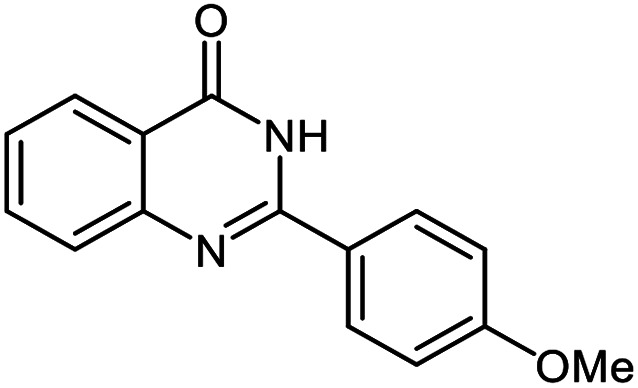	19	93	242–247 (ref. 21)
4	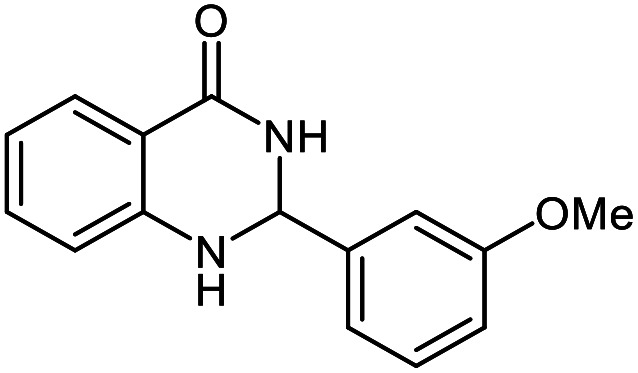	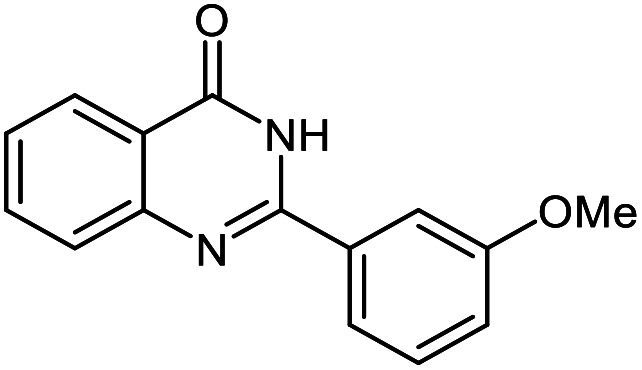	20	95	180–182 (ref. 21)
5	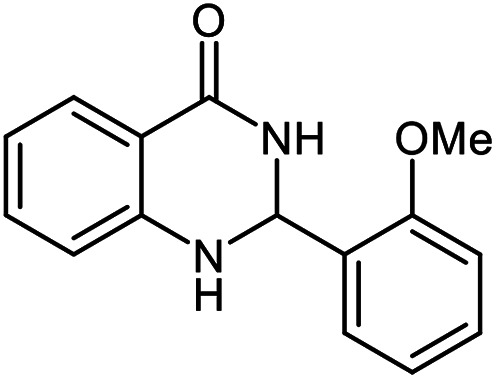	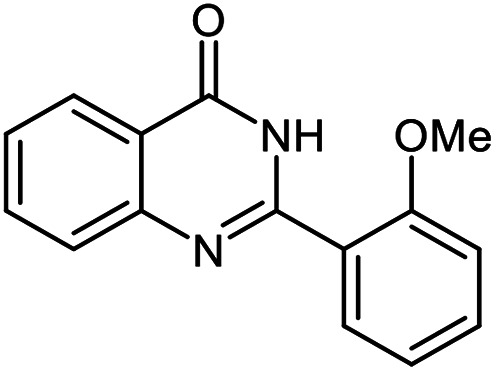	24	92	200–202 (ref. 21)
6	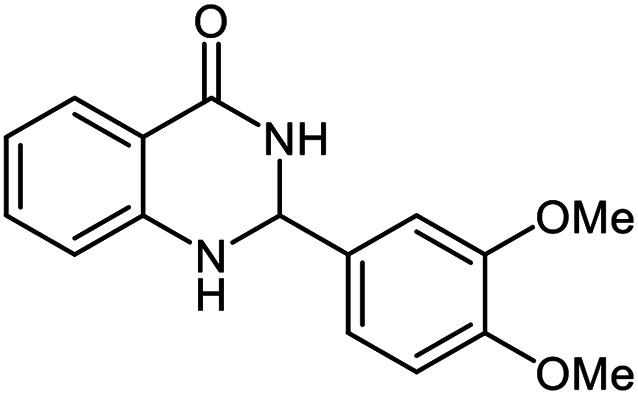	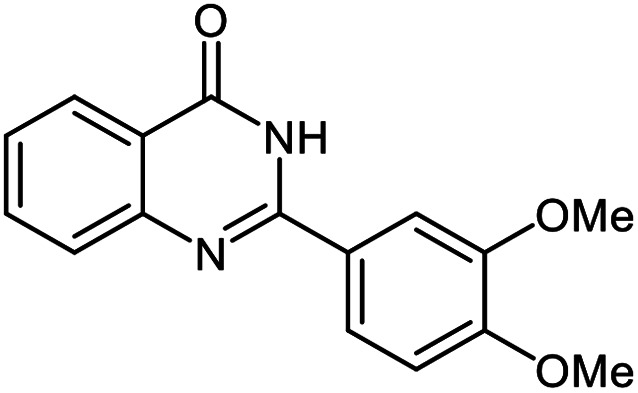	22	92	242–243 (ref. 22)
7	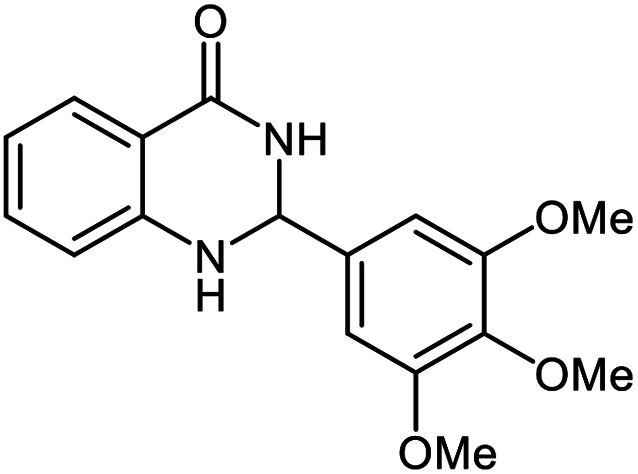	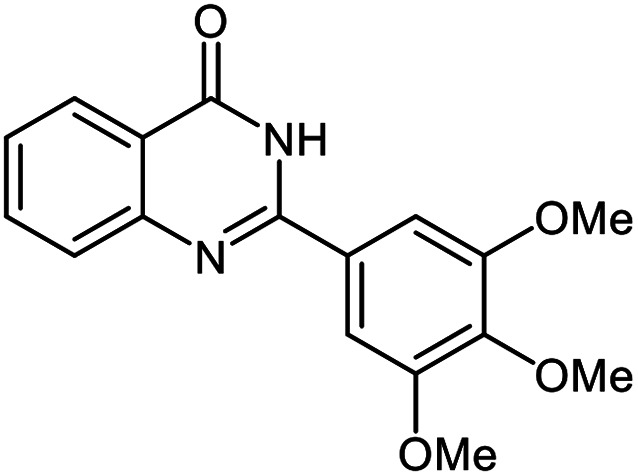	25	90	257–260 (ref. 21)
8	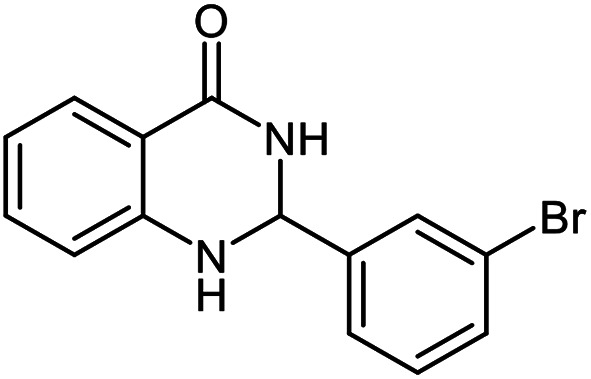	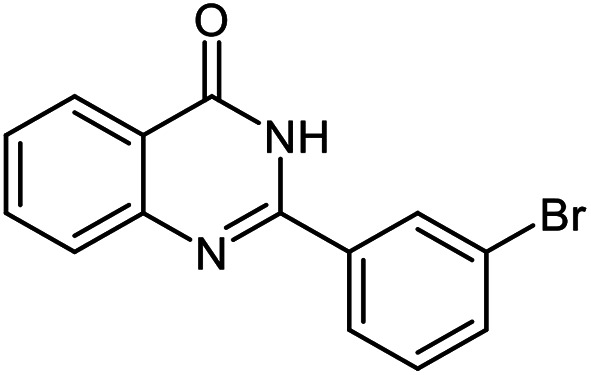	24	93	295–296 (ref. 22)
9^b^	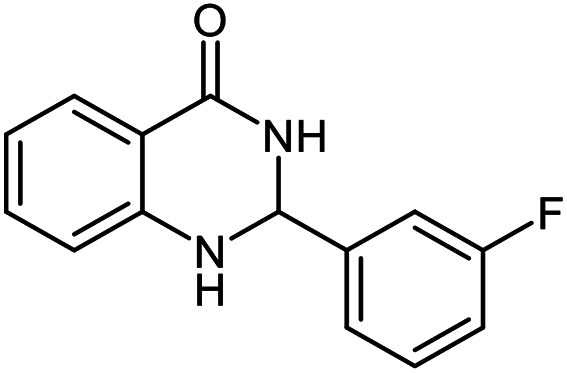	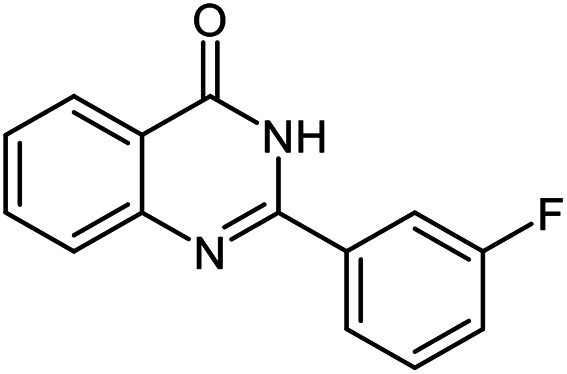	24	85	288–289
10^b^	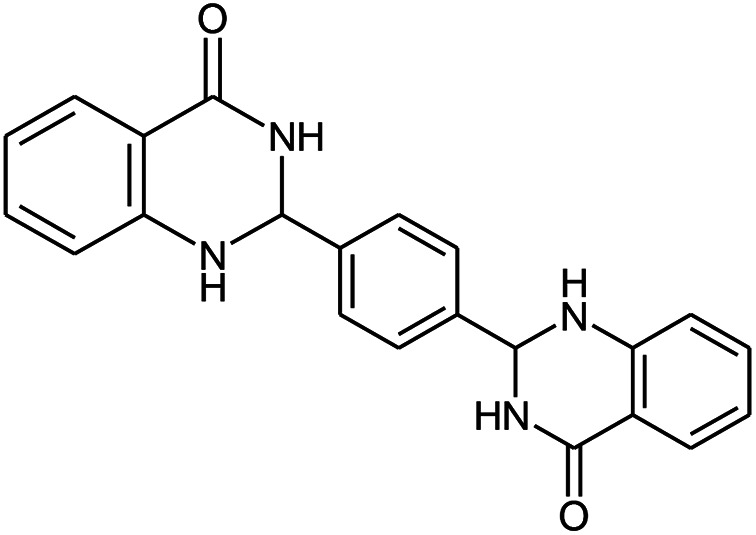	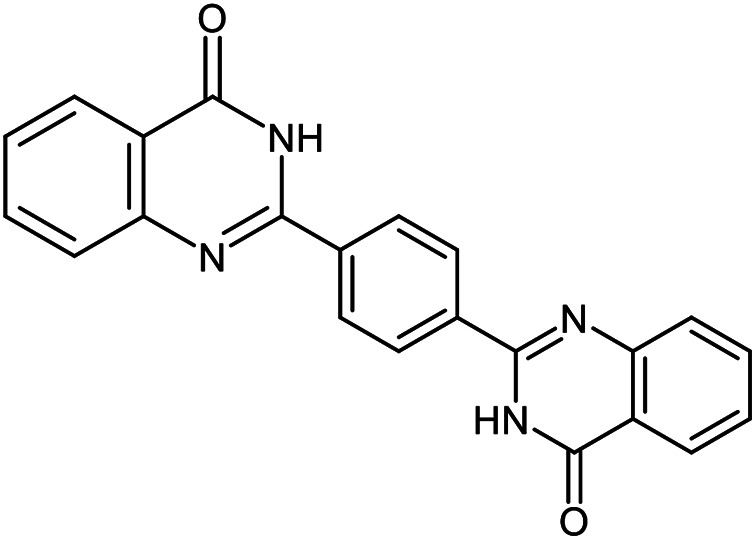	24	80	>300

a Reaction conditions: 2,3-dihydroquinazolin-4(1*H*)-one (1 mmol), laccase (200 U), DDQ (20 mol%), O_2_ (balloon), phosphate buffer (0.1 M, pH 4.5, 12.5 mL), MeCN (0.5 mL), 45 °C.

b The conversion was not 100%.

**Scheme 2:**
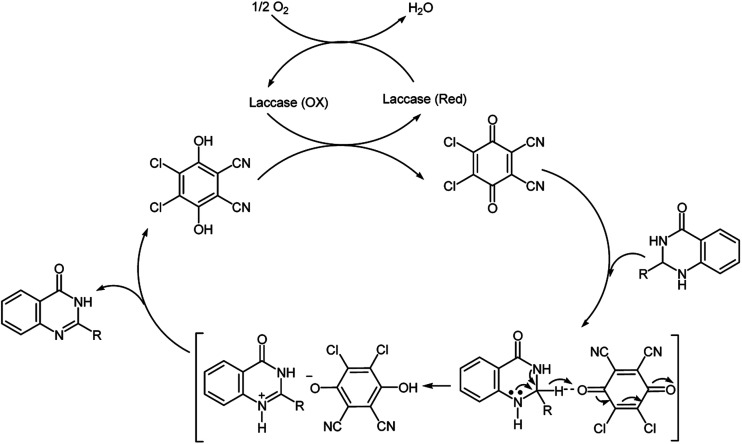
Proposed mechanism for the synthesis of quinazolinones using O_2_/laccase/DDQ system.

At this time the exact mechanisms of the reaction and the precise role of DDQ is not clear and should be further studied in detail. However, based on previously reported mechanisms for the application of DDQ as a hydride acceptor in dehydrogenation reactions^9,23^ and for oxidative dehydrogenation of *N*-heterocyclic compounds *via* an anomeric-based oxidation, a possible reaction pathway for the aerobic oxidation of dihydroquinazolinones to quinazolinones in the presence of laccase/DDQ cooperative catalyst system is suggested in Scheme 2. It is supposed that oxidation of the substrate occurs by hydride transfer from the substrate *via* anomeric-based oxidation^24^ to the DDQ, thereby forming an ion-pair adduct.^25^ Substrate-cation/DDQH-ion pair may convert to the desired product and DDQH_2_. Then, the by-product DDQH_2_ is oxidized by laccase leading to DDQ and reduced form of laccase.^20*d*^ Finally, the reduced laccase is reoxidized by molecular oxygen, consequently completing the catalytic cycle (Scheme 2).^19*c*^

The efficiency of O_2_/laccase/DDQ catalyst system was demonstrated by comparison our results on the oxidation of 2-phenyl 2,3-dihydroquinazolin-4(1*H*)-one with the previously reported methods (Table 3). A case study shows that the present protocol is superior than the other systems owing to of free from any toxic transition metal and halide, the use of O_2_ as a green, inexpensive and abundant oxidant and ambient temperature.

**Table 3:** Comparison of the synthesis of 2-phenyl quinazolin-4(3*H*)-one with previously reported methods

Entry	Reaction conditions	Time (h)	Isolated yield (%)	Ref.
1	DMSO, 100 °C	36	98	4
2	TBAB (1.6 mmol), CuCl_2_ (1.4 mmol), 100 °C	1.5	87	26
3	I_2_ (0.55 mmol), EtOH, 78 °C	6	99	27
4	MNPs-DABCO tribromide (50 mg), H_2_O_2_ (2.4 eq.), EtOH, 78 °C	9	90	24*b*
5	Laccase (200 U)/DDQ (20 mol%), NaPBS/CH_3_CN, O_2_ or air, 45 °C	24	90	Current study

In another effort, we examined the catalytic activity of O_2_/laccase/DDQ system for the synthesis of 2-arylbenzothiazoles *via* oxidative cyclization of Schiff bases derived from the condensation of 2-aminothiophenol with aldehydes. At the beginning, the reaction of 2-aminothiophenol with benzaldehyde was chosen as the model reaction. The examination of the different parameters such as the effects of the solvents, temperature and the amounts of laccase and DDQ on the model reaction revealed that 100 U of laccase, 0.1 mmol of DDQ under air in NaPBS (0.1 M, pH = 5) at room temperature is the best reaction conditions for complete conversion of starting materials to the desired product (Table 4, entry 6). It should be mentioned that the reaction was not completed under other reaction conditions shown in Table 4.

**Table 4:** Screening of the reaction conditions for the synthesis of 2-phenylbenzothiazole from oxidative cyclization of 2-aminothiophenol and benzaldehyde^a^

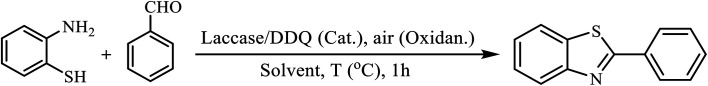						
Entry	Laccase (U)	DDQ (mol%)	Solvent	Temperature (°C)	pH	Isolated yield%
1	50	—	NaPBS	r.t.	5	30
2	50	5	NaPBS	r.t.	5	50
3	50	10	NaPBS	r.t.	5	70
4	50	20	NaPBS	r.t.	5	70
5	—	10	NaPBS	r.t.	5	40
6	100	10	NaPBS	r.t.	5	95^b^
7	100	10	MeCN/NaPBS	r.t.	5	80
8	100	10	EtOH/NaPBS	r.t.	5	60
9	100	10	MeOH/NaPBS	r.t.	5	70
10	100	10	THF/NaPBS	r.t.	5	40
11	100	10	NaPBS	60	5	60
11	100	10	NaPBS	r.t.	4	80
13	100	10	NaPBS	r.t.	6	90

a Reaction conditions unless stated otherwise: aldehyde (1 mmol), 2-aminothiopheno (1 mmol), air, solvent (12 mL), 1 h.

b The reaction was completed (conversion: 100%).

The scope of the reaction was extended to different aldehydes under optimized conditions (Table 5). As shown in Table 5, numerous aldehydes such as benzaldehydes with electron-donating and electron-withdrawing groups, heterocyclic and α,β-unsaturated aldehydes, 1-naphthaldehyde, 2-naphthaldehyde, 9-anthraldehyde, and terephthaldehyde were successfully applied to prepare the corresponding products *via* the reaction with 2-aminothiophenol. It should be mentioned that in some cases low amounts of starting material were observed together with the main product (Table 5, entries 5, 7, 18, 20–23) and in case of terephthaldehyde the trace amount of 4-(1,3-benzothiazol-2-yl) benzaldehyde was detected.

**Table 5:** Aerobic oxidative synthesis of benzothiazole derivatives using air/laccase/DDQ system^a^

Entry	Aldehyde	Product	Isolated yield%	Mp. (°C) (lit.)
1	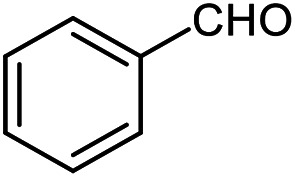	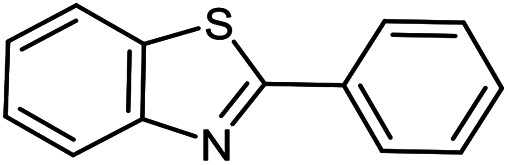	95	217–219 (ref. 28)
2	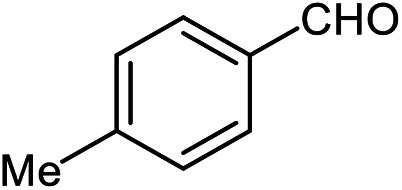	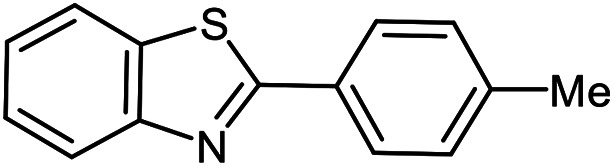	90	79–81 (ref. 28)
3	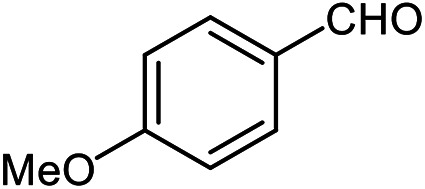	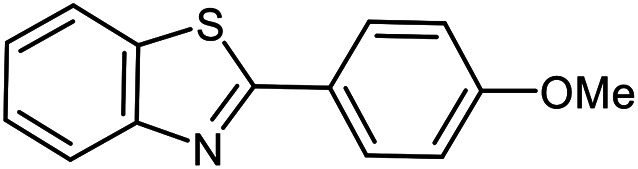	98	121–122 (ref. 28)
4	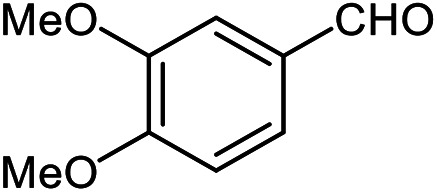	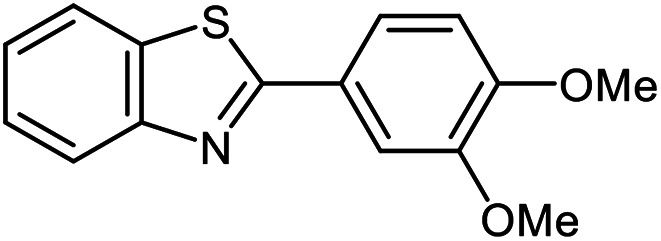	96	135 (ref. 28)
5^b^	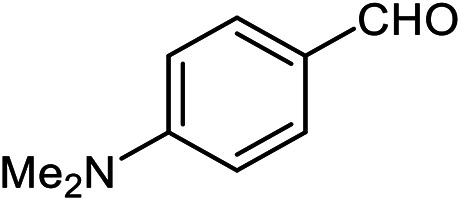	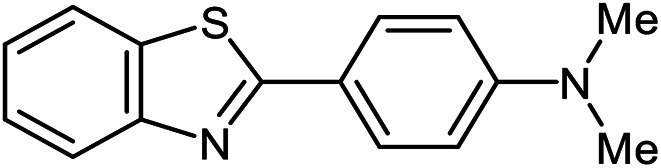	80	173–174 (ref. 28)
6	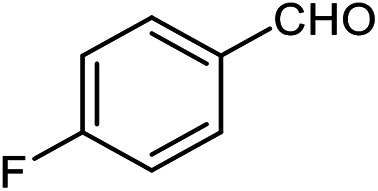	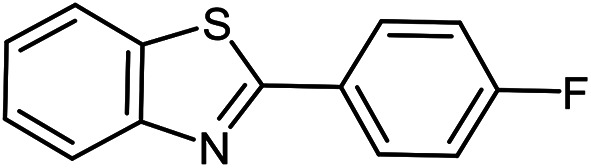	97	100–101 (ref. 28)
7	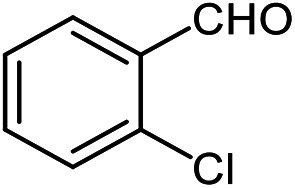	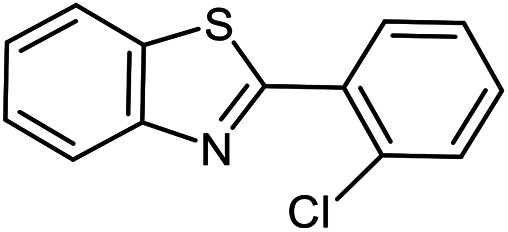	87	80–82 (ref. 28)
8	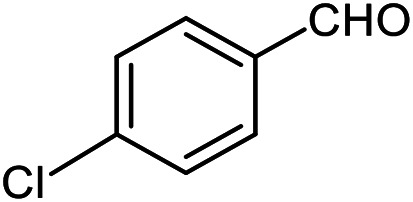	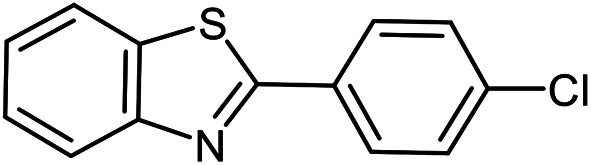	98	115–117 (ref. 28)
9	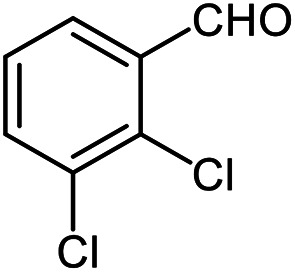	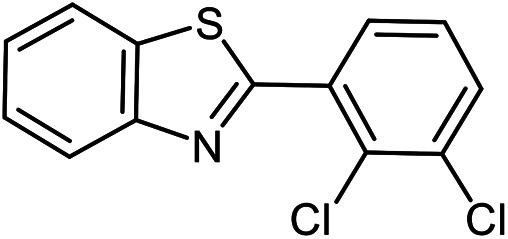	92	120–122 (ref. 29)
10	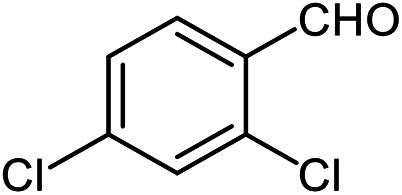	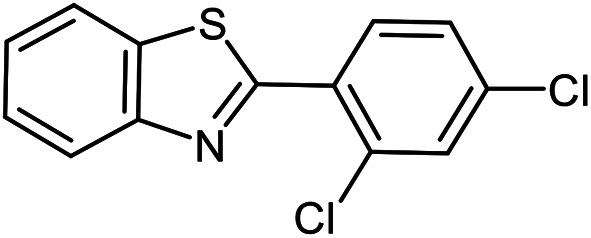	94	144 (ref. 30)
11	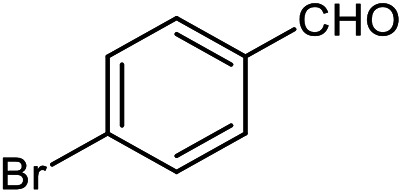	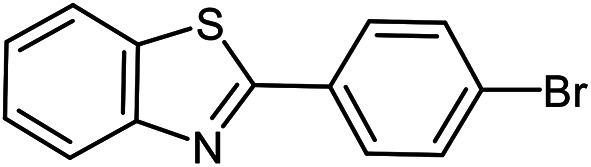	98	133 (ref. 28)
12	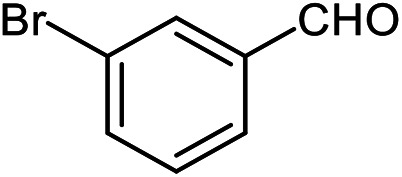	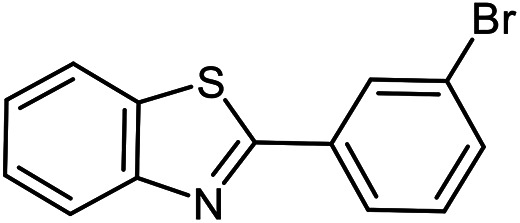	96	93–95 (ref. 31)
13	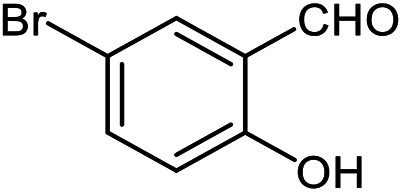	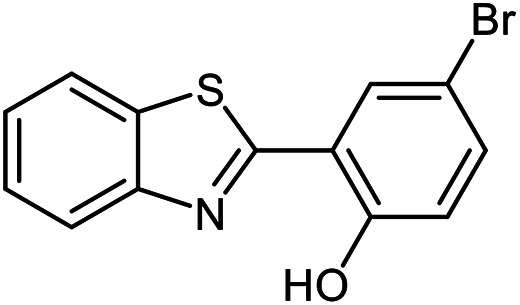	95	164 (ref. 28)
14	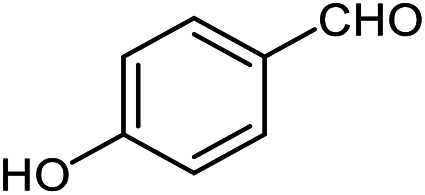	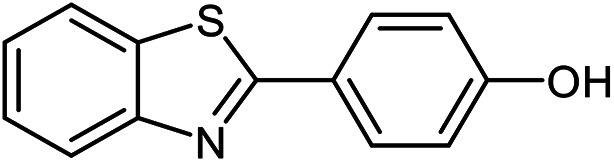	90	227–229 (ref. 28)
15	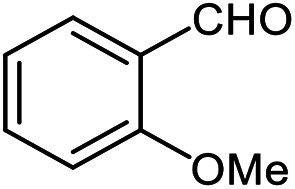	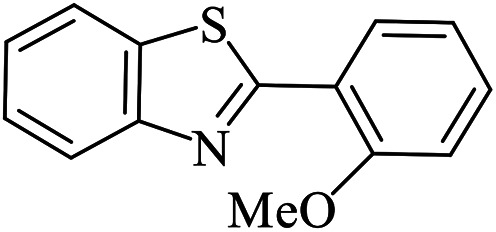	90	121–123 (ref. 32)
16	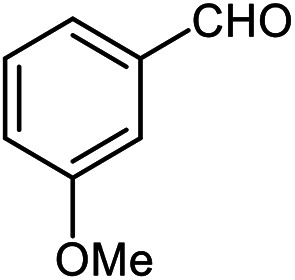	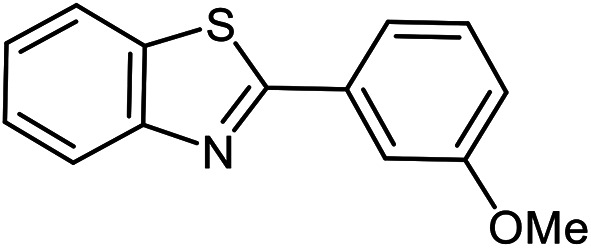	93	81–83 (ref. 33)
17	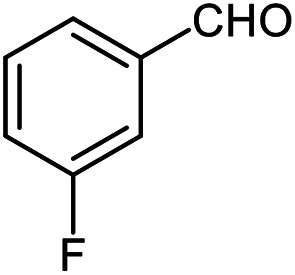	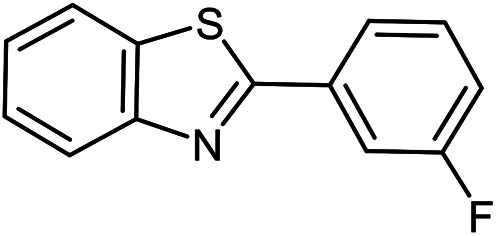	95	84–85 (ref. 31)
18^b^	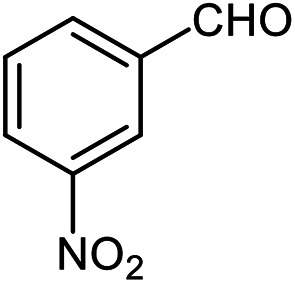	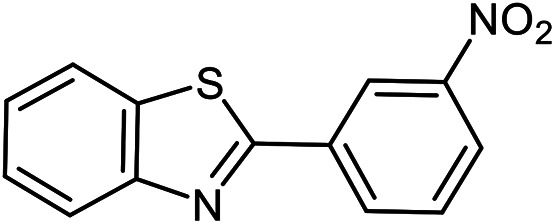	75	182–184 (ref. 29)
19	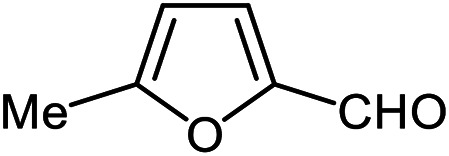	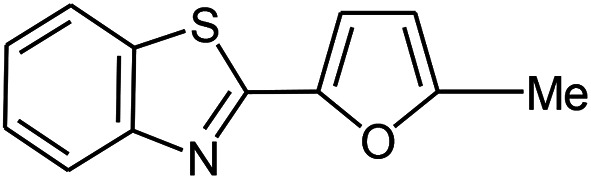	95	100–102 (ref. 34)
20^b^	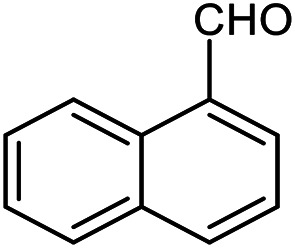	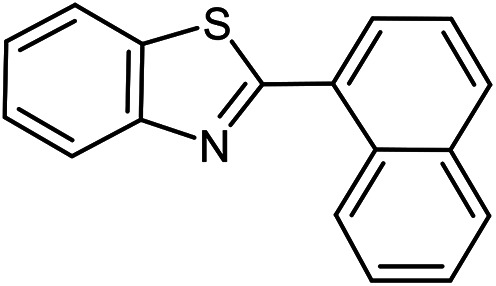	86	129–130 (ref. 31)
21^b^	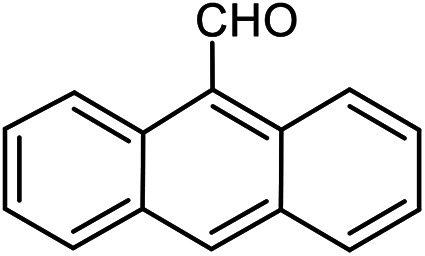	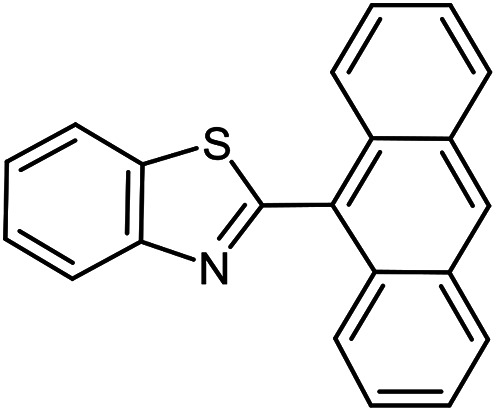	65	218–220 (ref. 28)
22^b^	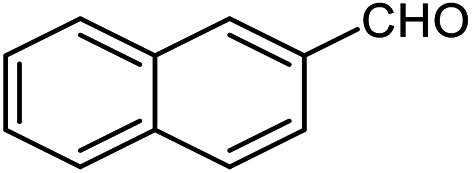	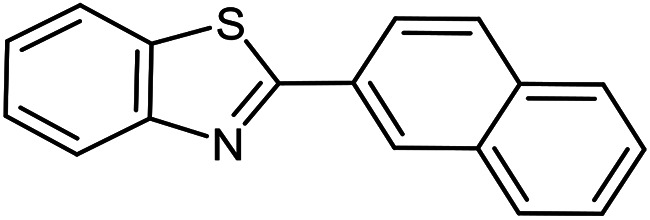	85	129–130 (ref. 28)
23^b^	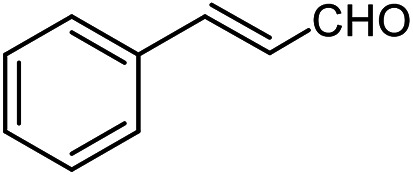	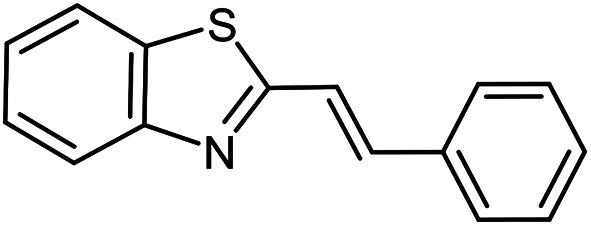	70	111 (ref. 35)
24^c^^,^^d^	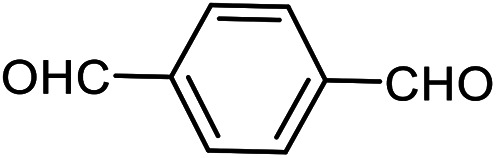	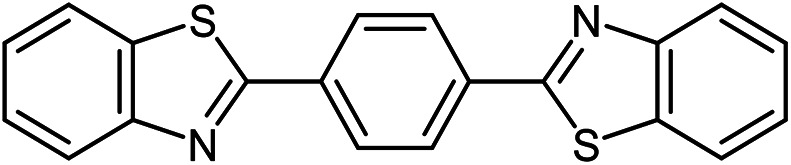	72	259–261 (ref. 28)

a Reaction conditions: 2-aminothiophenol (1 mmol), aldehyde (1 mmol), DDQ (10 mol%), laccase (87 mg, 100 U), phosphate buffer (0.1 M, 12 mL, pH = 5), r.t., 1 h.

b The conversion was not 100%.

c Reaction conditions: 2-aminothiophenol (2 mmol), aldehyde (1 mmol), DDQ (20 mol%), laccase (174 mg, 200 U), phosphate buffer (0.1 M, 12 mL, pH = 5), r.t., 1 h.

d The trace amount of 4-(1,3-benzothiazol-2-yl) benzaldehyde was detected.

## 3. Experimental

### 3.1. Determination of the activity of laccase from *Trametes versicolor*

The activity of commercial enzyme laccase from *Trametes versicolor* was determined *via* UV-Vis spectroscopy by monitoring the oxidation of 2,2′-azino-bis-(3-ethylbenzthiazoline-6-sulfonic acid) (ABTS, *ε* = 36 000 M^−1^ cm^−1^).^36^ One unit was defined as the amount of enzyme that oxidizes 1 μmol of ABTS per minute. The activity of the laccase enzyme batch applied in this investigation was evaluated at 0.87 U mg^−1^.

### 3.2. General procedure for the synthesis of 2,3-dihydroquinazolin-4(1*H*)-one derivatives

A mixture of an aromatic aldehyde (1 mmol) and *o*-anthranilamide (1 mmol) in water (2 mL) was stirred in the presence of sulfamic acid (0.01 g, 0.1 mmol) for 30 min at room temperature. The progress was monitored by TLC (*n*-hexane/EtOAC: 3/1). Upon completion, the solid product was isolated by filtration and washed with water. The crude product was recrystallized from ethanol to give the desired product.

### 3.3. General procedure for the synthesis of quinazolinone derivatives

A round-bottomed flask (25 mL) with a magnetic stir bar was charged with 2,3-dihydroquinazolin-4(1*H*)-one (1 mmol), DDQ (45.4 mg, 0.2 mmol) and acetonitrile (0.5 mL). Na-Phosphate buffer solution (NaPBS) (0.1 M, pH 4.5, 12.5 mL) and laccase from *Trametes versicolor* (200 U, 174 mg) were added and the reaction mixture was stirred under O_2_ (balloon) at 45 °C for the time given in Table 2. Then, the reaction mixture was extracted with EtOAc (3 × 10 mL), and the organic layer was dried over anhydrous Na_2_SO_4_ and evaporated under reduced pressure. Finally, the crude product was purified by column chromatography on SiO_2_ using *n*-hexane/ethyl acetate (75 : 25).

### 3.4. General procedure for the synthesis of benzothiazole derivatives

In an open-air round-bottom flask (25 mL) equipped with a magnetic stirrer, a mixture of 2-aminothiophenol (1 mmol) and aldehyde (1 mmol) in phosphate buffer (0.1 M, 12 mL, pH = 5) was prepared. Then, DDQ (10 mol%) and laccase (87 mg, 100 U) were added to the reaction mixture and stirred at room temperature for 1 h. The product was extracted with EtOAc (3 × 10 mL) and the combined organic phases were dried over anhydrous Na_2_SO_4_. The evaporation of the solvent and purification by column chromatography (*n*-hexane/EtOAc) gave the desired product.

## 4. Conclusions

In summary, laccase/DDQ was applied as a bioinspired cooperative catalytic system for the aerobic oxidative synthesis of quinazolinones and benzothiazoles. The significant advantages of these methods are as follows: (i) the use of air or O_2_ as an environmentally benign, inexpensive and abundant oxidant and the formation of H_2_O as the only nontoxic by-product; (ii) the synthesis of structurally diverse quinazolinones and benzothiazoles in good to high yields in aqueous media at ambient temperature; (iii) the present methods are superior to other currently available and attractive for pharmaceutical industry owing to free from any toxic and expensive transition metals and halides catalysts; (iv) these methods conform to several of the guiding principles of green chemistry.

We believe that the results presented here open up a new avenue for application of laccase/DDQ catalyst system to accomplish other green and sustainable synthetic transformations.

## Conflicts of interest

There are no conflicts to declare.

## Supplementary Material

RA-010-C9RA10303A-s001
