# Supplementary material for: Aerobic oxidative synthesis of quinazolinones and benzothiazoles in the presence of laccase/DDQ as a bioinspired cooperative catalytic system under mild conditions
Source: RSC Adv. 2020 Apr 8;10(24):14254–61. doi: 10.1039/c9ra10303a (PMC9051882; doi:10.1039/c9ra10303a)
Supplement: RA-010-C9RA10303A-s001 [file RA-010-C9RA10303A-s001.pdf]

Electronic Supplementary Material (ESI) for RSC Advances.
This journal is © The Royal Society of Chemistry 2020

Supplementry information

Aerobic Oxidative Synthesis of Quinazolinones and Benzothiazoles in the Presence of Laccase/DDQ as a Bioinspired Cooperative Catalytic System under Mild Conditions

Nadia Ghorishi, Zahra Shokri, Reza Moradi, Amira Abdelrasoul, Amin Rostami*

1. General Information

All chemicals and solvents were bought from the Merck and Aldrich Chemical Companies and used without further purification. Laccase from Trametes versicolor (CAS Number: 0080498153, Product Number: 38429-10G) was purchased from Sigma-Aldrich (St. Louis, MO, USA). Melting points were obtained on an Electrothermal 9100 apparatus and are uncorrected. ^{1}H NMR spectra were recorded on BrukerAvance spectrometer (250 and 400 MHz).

2. Analytical data of the products

2-Phenylquinazolin-4(3*H*)-one (Table 2, entry 1):[1] R_f = 0.50 (*n*-hexane/ethyl acetate: 3:1), ^{1}H NMR (DMSO-d$_6$, 250 MHz): δ (ppm)= 12.49 (s, 1H), 8.16-8.14 (m, 3H), 7.80-7.69 (m, 2H), 7.54-7.49 (m, 4H).

2-(4-Methylphenyl)quinazolin-4(3*H*)-one (Table 2, enrty 2):[1] R_f = 0.53 (*n*-hexane/ethyl acetate: 3:1), ^{1}H NMR (DMSO-d$_6$, 250 MHz): δ (ppm)= 12.38 (s, 1H), 8.18-8.09 (m, 3H), 7.79 (t, *J* = 7.2 Hz, 1H), 7.69 (d, *J* = 8.0 Hz, 1H), 7.45 (t, *J* = 7.2 Hz, 1H), 7.06 (d, *J* = 8.5 Hz, 2H), 3.82 (s, 3H).

2-(3-Methoxyphenyl)quinazolin-4(3*H*)-one (Table 2, entry 4):[1] R_f = 0.55 (*n*-hexane/ethyl acetate: 3:1), ^{1}H NMR (250 MHz, DMSO-d6): δ (ppm)= 12.53 (s, 1H), 8.14 (d, *J* = 7.7 Hz, 1H), 7.83-7.74 (m, 4H), 7.54-7.42 (m, 2H), 7.14 (d, *J* = 8.0 Hz, 1H), 3.85 (s, 3H).

2-(2-Methoxyphenyl)quinazolin-4(3*H*)-one (Table 2, entry 5):[1] R_f = 0.55 (*n*-hexane/ethyl acetate: 3:1), ^{1}H NMR (250 MHz, DMSO-d6): δ (ppm)= 12.10 (s, 1H), 8.13 (d, *J* = 7.5 Hz, 1H), 7.81-7.52 (m, 5H), 7.18 (d, *J* = 8.0 Hz, 1H), 7.10-7.07 (m, 1H), 3.84 (s, 3H).

2-(3-Bromophenyl)quinazolin-4(3*H*)-one (Table 2, entry 8):[2] R_f = 0.60 (*n*-hexane/ethyl acetate: 3:1), ^{1}H NMR (DMSO-d$_6$, 400 MHz): δ (ppm)= 12.62 (br s, 1H), 8.37(s, 1H), 8.20 (d, *J* = 7.5 Hz, 1H), 8.17 (d, *J* = 7.5 Hz, 1H), 7.88-7.76 (m, 3H), 7.57-7.50 (m, 2H).

2-(3-Fluorophenyl)quinazolin-4(3*H*)-one (Table 2, entry 9): R_f = 0.6 (*n*-hexane/ethyl acetate: 3:1), ^{1}H NMR (DMSO-d$_6$, 400 MHz): δ (ppm)= 12.60 (s, 1H), 8.15 (d, *J* = 7.7 Hz, 1H), 8.06-7.97 (m, 2H), 7.86-7.72 (m, 2H), 7.63-7.40 (m, 3H).

2-phenylbenzothiazole (Table 5, entry 1):[3] R_f = 0.60 (*n*-hexane/ethyl acetate: 2:1), ^{1}H NMR ($CDCl_3$, 250 MHz): δ (ppm) = 8.10-8.08 (m, 3H), 7.91 (d, 1H, *J* = 7.5 Hz), 7.50-7.49 (m, 4H), 7.42-7.36 (m, 1H).

2-(4-fluorophenyl)benzothiazole (Table 5, entry 6):[3] R_f = 0.65 (*n*-hexane/ethyl acetate: 2:1), ^{1}H NMR ($CDCl_3$, 250 MHz): δ (ppm)= 8.12-8.05 (m, 3H), 7.90 (d, 1H, *J* = 7.5 Hz), 7.50 (t, 1H, *J* = 7.2 Hz), 7.39 (t, 1H, *J* = 7.2 Hz), 7.26-7.15 (m, 2H).

2-(benzothiazol-2-yl)-4-bromophenol (Table 5, entry 13): R_f = 0.55 (*n*-hexane/ethyl acetate: 2:1), ^{1}H NMR ($CDCl_3$, 250 MHz): δ (ppm)= 12-13 (s, 1H), 7.94 (dd, 2H, *J* =19.5, 8.0 Hz), 7.56 (d, 1H, *J* = 2.0 Hz), 7.54-7.41 (m, 3H), 6.98 (d, 1H, *J* = 8.8 Hz).

2-(3-nitrophenyl)benzothiazole (Table 5, entry 18):[3] R_f = 0.7 (*n*-hexane/ethyl acetate: 2:1), ^{1}H NMR ($CDCl_3$, 250 MHz): δ (ppm)= 8.93 (s, 1H), 8.43 (d, 1H, *J* = 7.7 Hz), 8.34 (d, 1H, *J* = 8.0 Hz), 8.12 (d, 1H, *J* = 7.7 Hz), 7.96 (d, 1H, *J* = 7.7 Hz), 7.70 (t, 1H, *J* = 8.0 Hz), 7.55-7.42 (m, 2H).

2-(5-methylfuran-2-yl)benzothiazole (Table 5, entry 19): R_f = 0.6 (*n*-hexane/ethyl acetate: 2:1), ^{1}H NMR ($CDCl_3$, 250 MHz): δ (ppm)= 8.03 (d, 1H, *J* = 8.2 Hz), 7.86 (d, 1H, *J* = 8.0 Hz), 7.46 (m, 1H), 7.34 (m,1H), 7.07 (d, 1H, *J* = 3.0 Hz), 6.18 (d, 1H, J = 3.0 Hz), 2.43 (s, 3H).

2-(naphthalen-1-yl)benzothiazole (Table 5, entry 20):[3] R_f = 0.6 (*n*-hexane/ethyl acetate: 2:1), ^{1}H NMR ($CDCl_3$, 250 MHz): δ (ppm)= 8.57 (br s, 1H), 8.21 (d, 1H, *J* = 7.2 Hz), 8.12 (d, 1H, *J* = 8 Hz), 7.97-7.92 (m, 4H), 7.57-7.49 (m, 4H), 7.43-7.37 (m, 1H).

3. ^{1}H NMR of the products

^{1}HNMR (Table 2, entry 1)

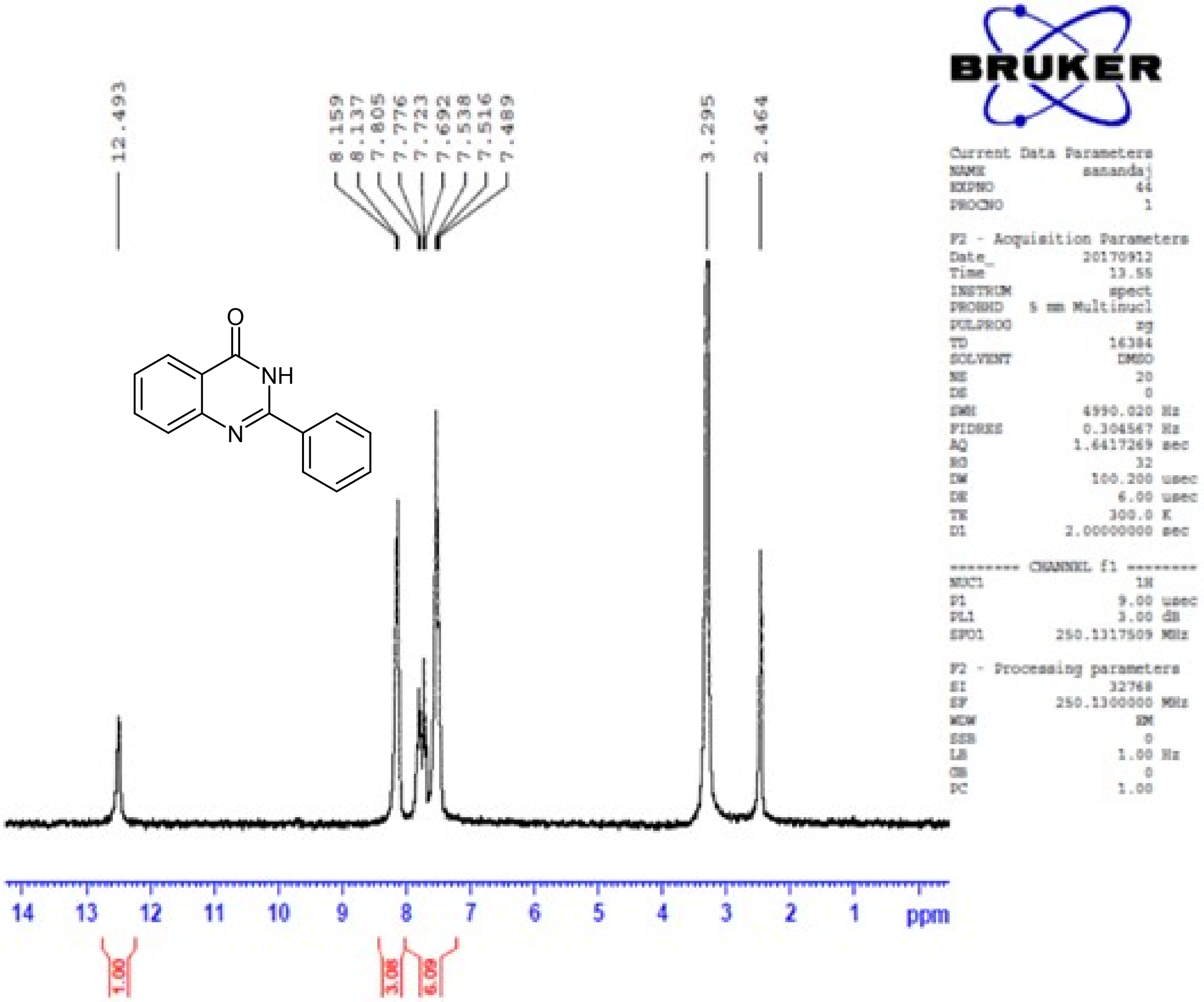

¹HNMR (Table 2, entry 2)

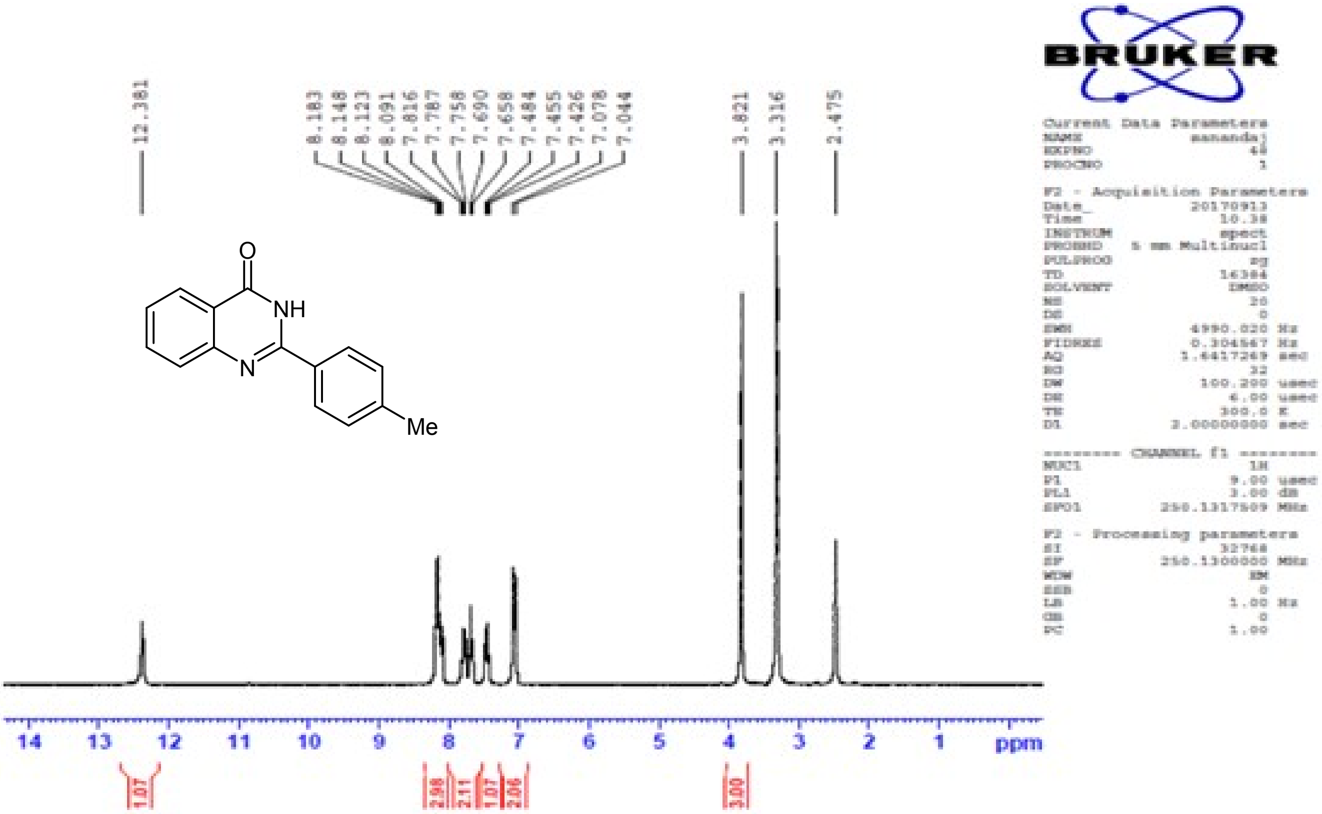

¹HNMR (Table 2, entry 4)

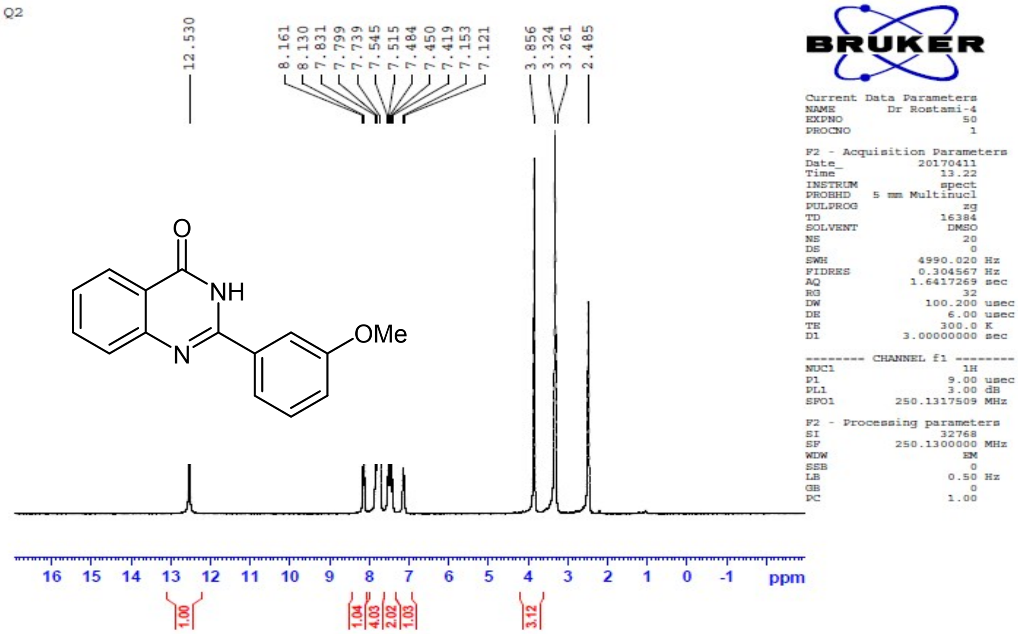

^{1}HNMR (Table 2, entry 5)

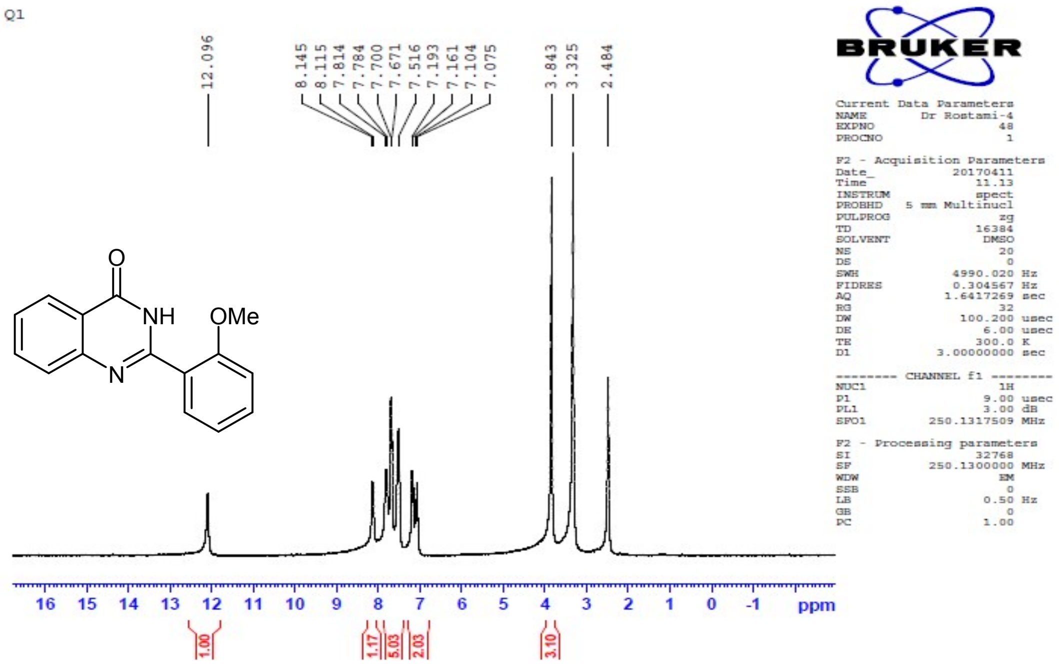

^{1}HNMR (Table 2, entry 8)

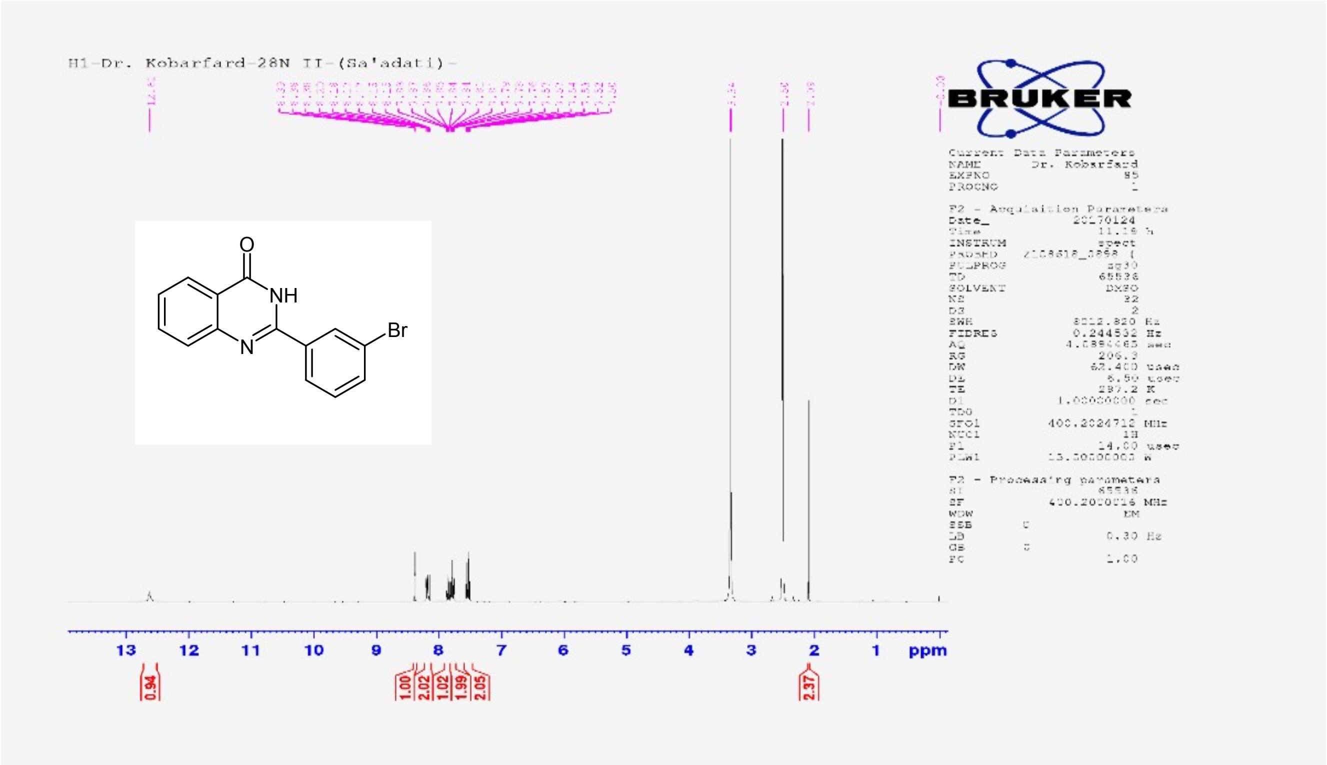

^{1}HNMR (Table 2, entry 9)

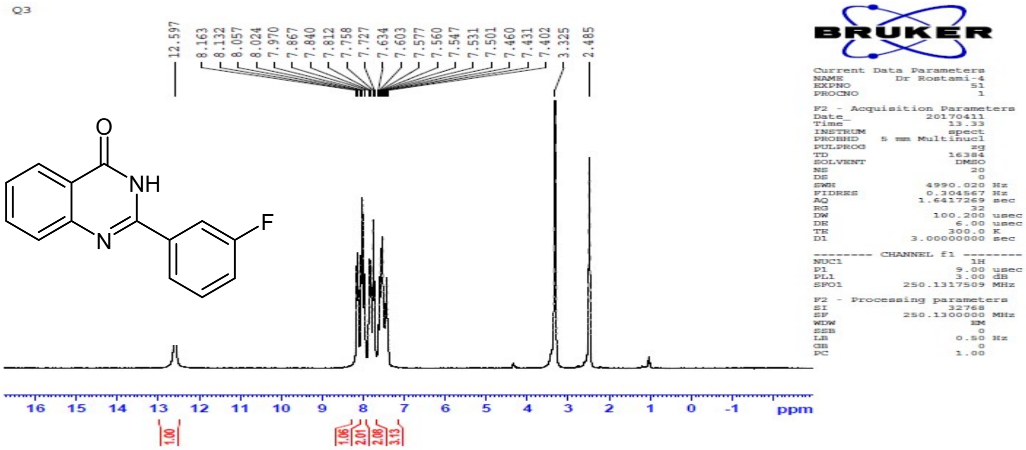

^{1}HNMR (Table 5, entry 1)

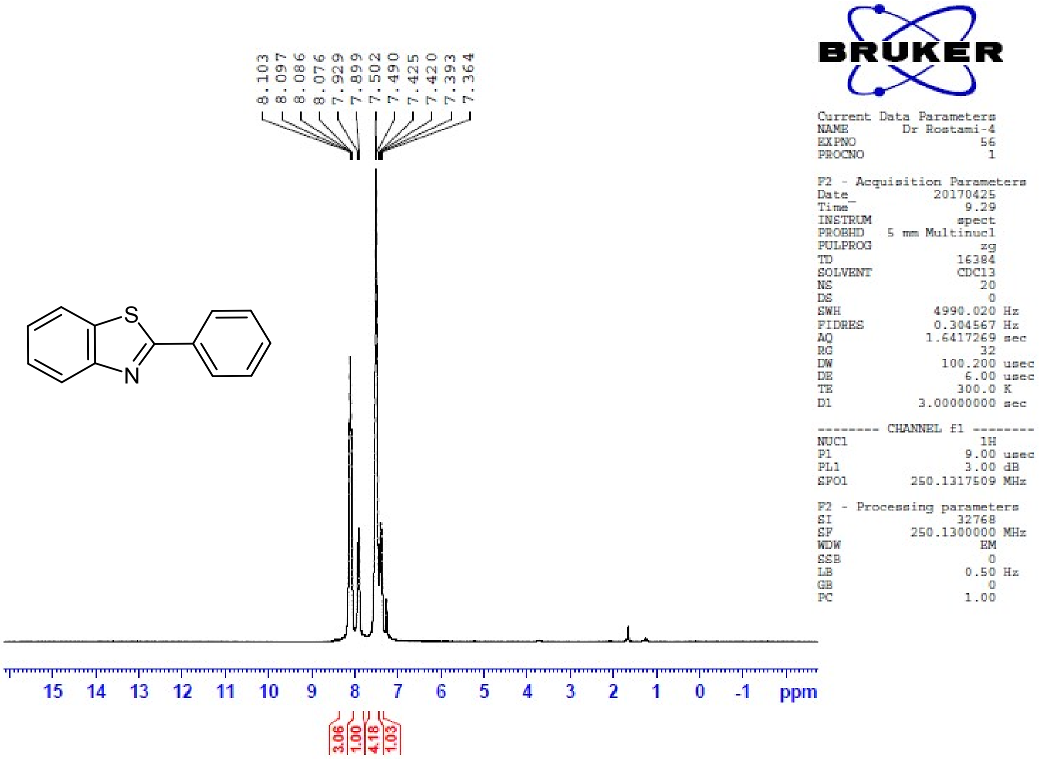

^{1}HNMR (Table 5, entry 6)

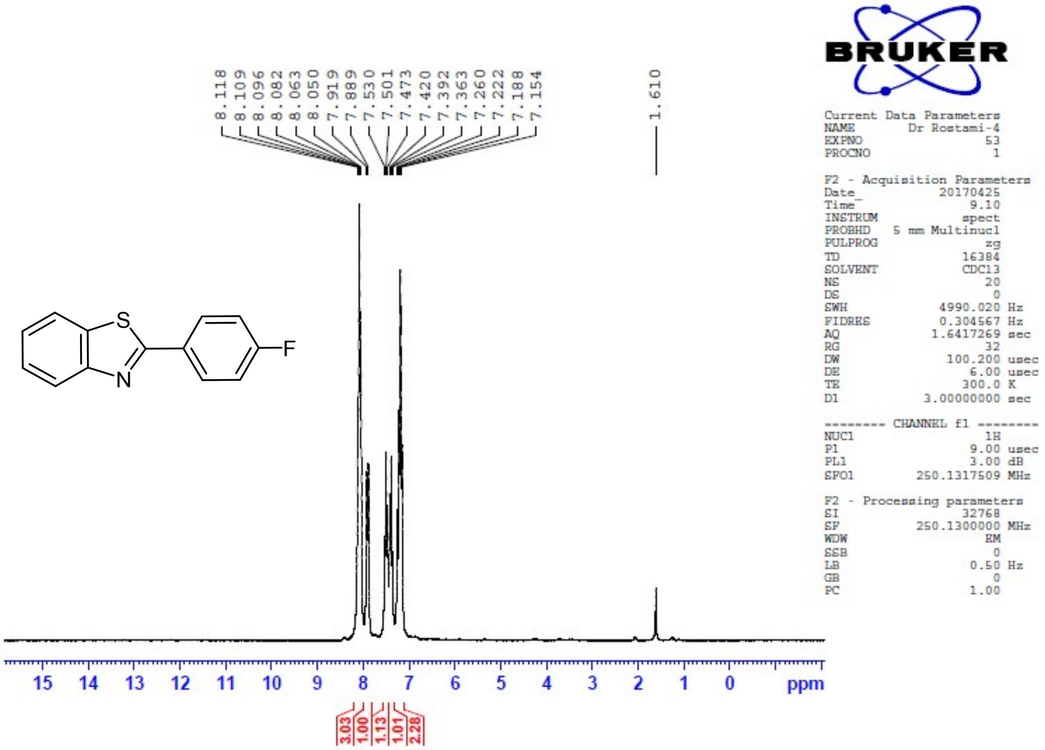

^{1}HNMR (Table 5, entry 13)

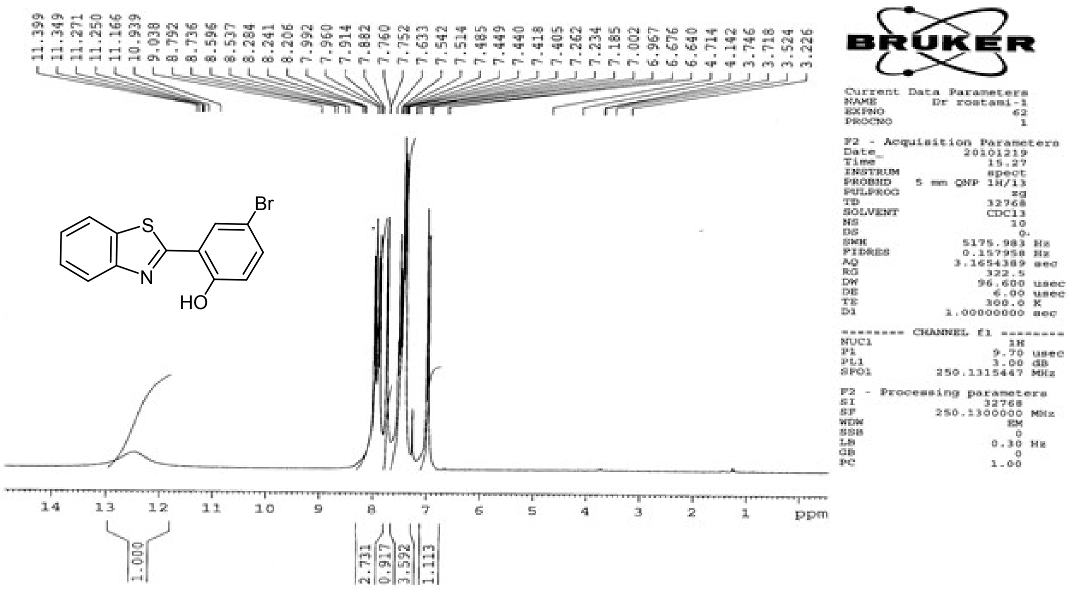

^{1}HNMR (Table 5, entry 18)

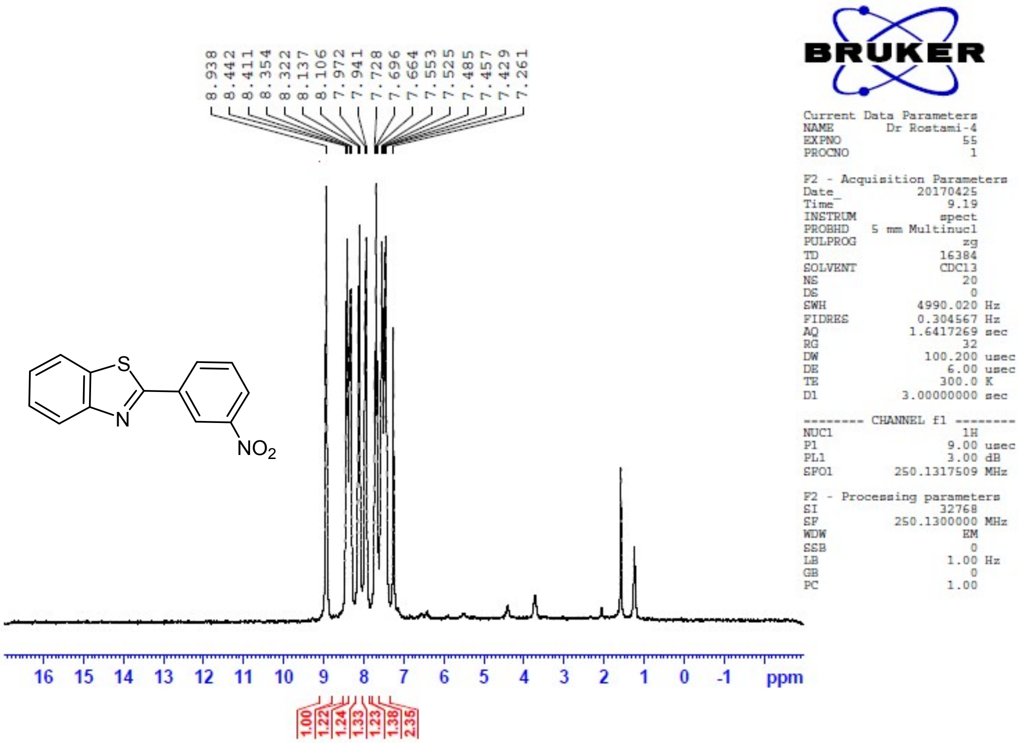

^{1}HNMR (Table 5, entry 19)

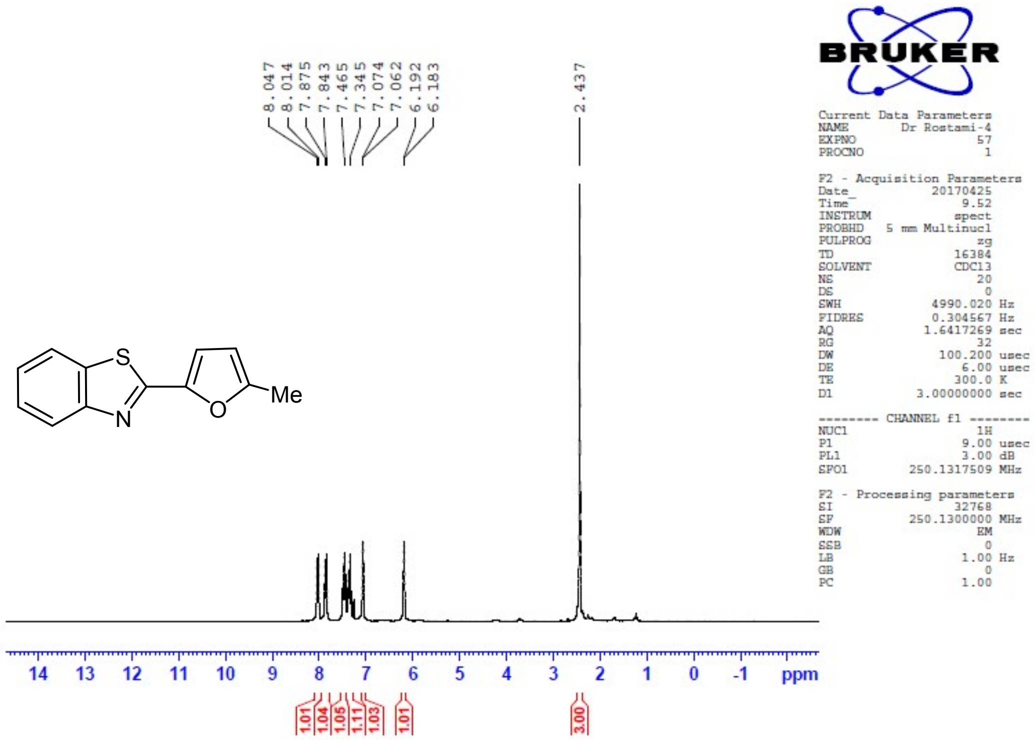

^{1}HNMR (Table 5, entry 20)

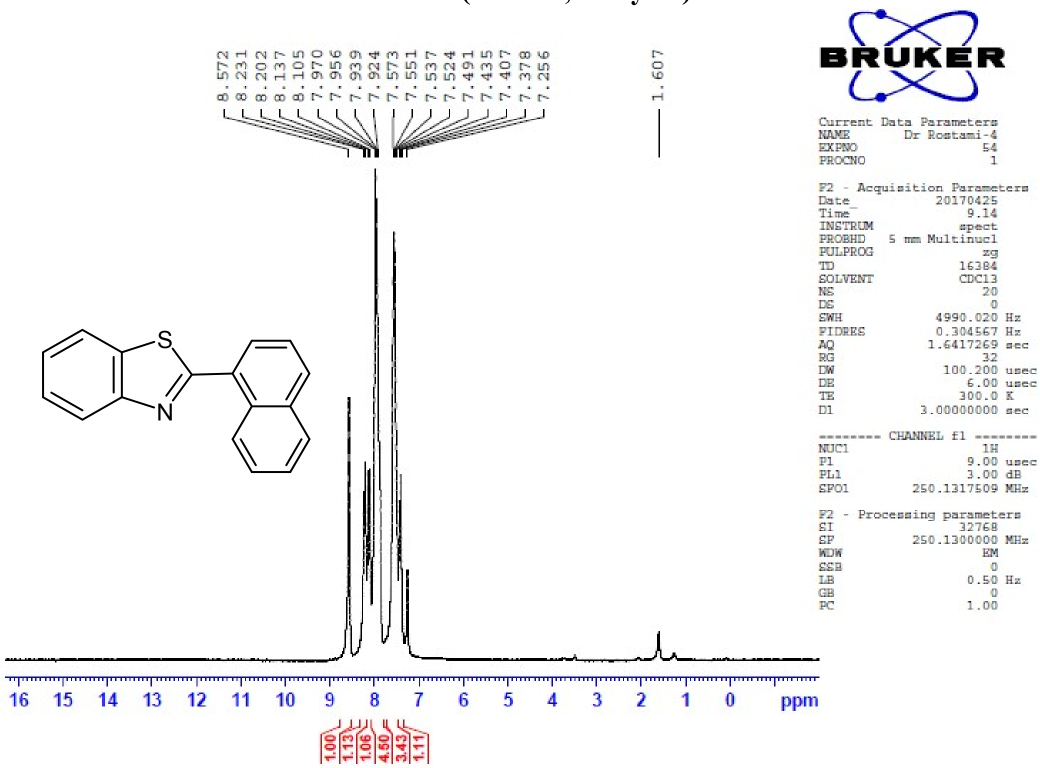

References

1. K. Upadhyaya, R. Kumar, T. Sanjeev, K. Shukla, R. Pati Tripathi, *J. Org. Chem.*, 2016, **81**, 5046.
2. J. Fang, J. Zhou, *J. Org. Chem.*, 2011, **76**, 7730.
3. X. L. Yang, C. M. Xu, S. M. Lin, J. X. Chen, J. C. Ding, H. Y. Wu, W. K. Sua, *J. Braz. Chem. Soc.*, 2010, **21**, 37.
